# The influence of antidepressant and psychotherapy treatment adherence on future work leaves for patients with major depressive disorder

**DOI:** 10.1186/s12888-020-02731-9

**Published:** 2020-06-19

**Authors:** Fraser W. Gaspar, Kerri Wizner, Joshua Morrison, Carolyn S. Dewa

**Affiliations:** 1 MDGuidelines, ReedGroup Ltd., Westminster, CO USA; 2 Department of Psychiatry and Behavioral Sciences, University of California at Davis, Davis, California USA

**Keywords:** Mental health, Short-term disability, Guidelines, Absence, Medication, Psychiatric services, Risk prediction

## Abstract

**Background:**

Depression is the greatest contributor to worldwide disability. The purpose of this study was to understand the influence of antidepressant and psychotherapy treatment adherence on future work leaves for patients with major depressive disorder.

**Methods:**

Patients with a newly diagnosed major depressive disorder (*n* = 26,256) were identified in IBM® Watson™ MarketScan® medical and disability claims databases. Antidepressant and psychotherapy adherence metrics were evaluated in the acute phase of treatment, defined as the 114 days following the depression diagnosis. Multiple variable Cox proportional hazards regression models evaluated the influence of antidepressant and/or psychotherapy adherence on future injury or illness work leaves.

**Results:**

The majority of work leaves in the 2-year follow-up period occurred in the acute phase of treatment (71.2%). Among patients without a work leave in the acute phase and who received antidepressants and/or psychotherapy (*n* = 19,994), those who were adherent to antidepressant or psychotherapy treatment in the acute phase had a 16% (HR = 0.84, 95% CI = 0.77–0.91) reduced risk of a future work leave compared to treatment non-adherent patients. Patients who were non-adherent or adherent to antidepressant treatment had a 22% (HR = 1.22, 95% CI = 1.11–1.35) and 13% (HR = 1.13, 95% CI = 1.01–1.27) greater risk of a future work leave, respectively, than patients not receiving antidepressant treatment. Conversely, patients who were non-adherent or adherent to psychotherapy treatment had a 9% (HR = 0.91, 95% CI = 0.81–1.02) and 28% (HR = 0.72, 95% CI = 0.64–0.82) reduced risk of a future work leave, respectively, than patients not receiving psychotherapy treatment.

**Conclusions:**

This analysis suggests that treatment adherence may reduce the likelihood of a future work leave for patients with newly diagnosed major depressive disorder. Psychotherapy appears more effective than antidepressants in reducing the risk of a future work leave.

## Background

More than half of household heads in the United States (U.S.) will experience work impairment due to a health condition in their lifetime [1]. Work impairments may cause reduced work productivity, negatively affect work schedule, or cause increased temporary work leaves. Workers with a work impairment lose immediate and future earnings [2]. Further, because employees with temporary disabilities account for the majority of healthcare and disability benefit payments, employers and health care payers bear a large financial burden from work impairments [3].

In 2016, 19% of years lived with a disability were due to mental disorders [4]. Not only are mental disorders one of the greatest contributors to work impairment with both high incidence of work loss and total lost work days [5], but mental illnesses are also risk factors for injury and illness work leaves not directly related to mental illness [6, 7]. Major depressive disorder (MDD) is one of the most common mental disorders. In 2018, 17.7 million U.S. adults (7.2% of the population) self-reported at least one major depressive episode in the past year [8]. Both antidepressant and psychotherapy treatment have been shown to be effective for patients with depression [9, 10]; however, treatment utilization is low with more than one-third of adults with MDD not receiving any treatment [11, 12]. In addition, when treatment is initiated, less than half of patients are adherent to guideline recommendations for antidepressant treatment [13, 14].

Despite the societal impact of MDD on lost work time through decreased functional capacity, limited research exists on whether depression treatments are effective at preventing a future work leave. To date, only one study has investigated how antidepressant treatment influenced future work leaves [15]. Burton et al. (2007) found that patients who were adherent to antidepressant medication had a lower probability of a future work leave compared with non-adherent patients. However, Burton et al. (2007) only investigated patients initiating antidepressant treatment and did not assess patients with psychotherapy treatment, despite psychotherapy treatment being the primary treatment modality for MDD patients [14].

The objective of this research was to use medical and disability claim databases to test the hypothesis that antidepressant and psychotherapy treatment adherence decrease the risk of a future work leave for patients with MDD.

## Methods

### Study data

This retrospective study analyzed claims from the IBM® Watson™ MarketScan® Commercial Claims and Encounters (CCAE) database and the MarketScan® Health and Productivity Management (HPM) database [16]. Claims were included from 2007 to 2017. The data were acquired through a license agreement for data use. Over 260 employers and 40 health plans, representing 350 unique carriers, contribute data to the MarketScan® databases. The MarketScan databases are a convenience sample of employees with employer-provided health insurance, mostly from large employers.

The CCAE database includes de-identified, patient-level outpatient, prescription, and inpatient information. The HPM database includes de-identified, employee-level disability leave information. The CCAE and HPM databases are linked via a unique identifier. This research is not considered human subject research as it does not involve interactions with – or interventions among – participants, and only de-identified data was available [17]. This research was not reviewed by an institutional review board; approval by an institutional review board was not needed because MarketScan® data are de-identified and comply with the Health Insurance Portability and Accountability Act.

**Fig. 1:**
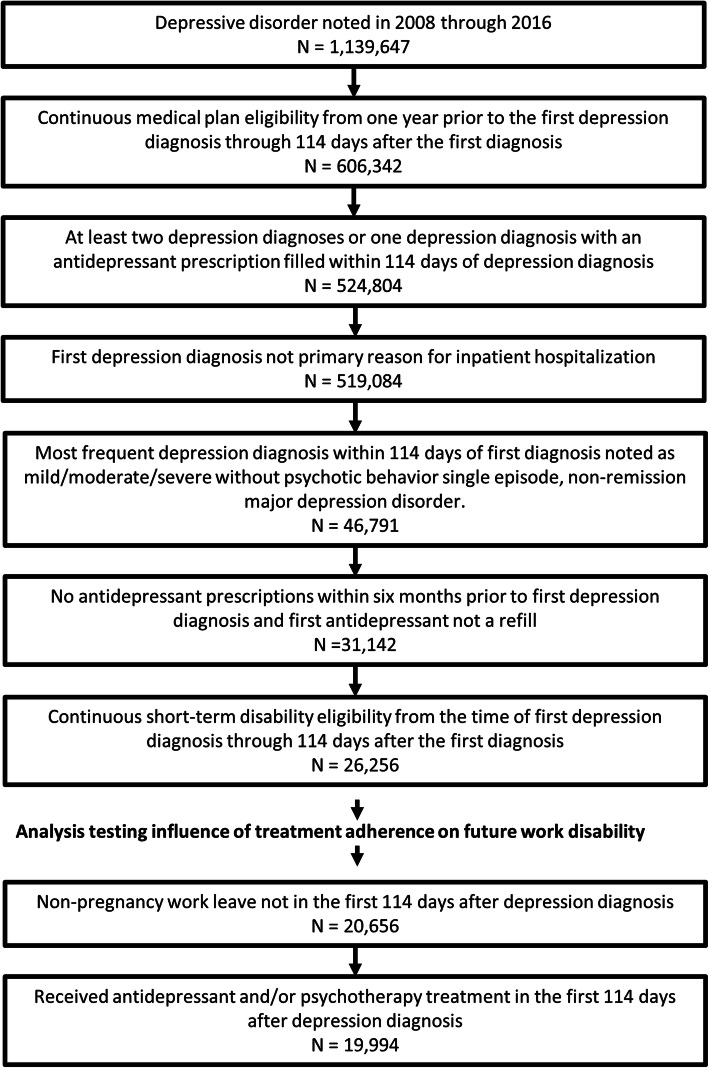
Flow chart depicting study population inclusion criteria

The study population was defined by identifying patients whose first depressive disorder diagnosis was recorded between January 1, 2008 and December 31, 2016 (Fig. 1). The MarketScan® databases code diagnoses using the International Classification of Diseases, Ninth Revision, Clinical Modification (ICD-9-CM) or the International Classification of Diseases, Tenth Revision, Clinical Modification (ICD-10-CM). Depression diagnoses were identified using the Agency for Healthcare Research and Quality’s Clinical Classification Software (CCS)‘s ICD-9-CM/ICD-10-CM diagnosis grouper for “depressive disorder” [18]. Patients were included if they had health care coverage for 1 year before and at least 114 days after their first reported depression diagnosis. The 1 year of health care coverage before diagnosis was used to identify newly diagnosed patients, versus previously diagnosed patients continuing their various treatment paths. The 114 days after the depression diagnosis, defined in this study as the acute phase of treatment [19], was used to understand the influence of treatment on future work leaves.

To help reduce the number of false-positives, patients were only included if their medical claims in the acute phase had either 1) two or more depression diagnoses or 2) one depression diagnosis with an antidepressant prescription filled in the acute phase of treatment [20]. Next, patients were included if the most frequent depressive disorder diagnosis in the acute phase was a mild, moderate, or severe without psychotic behavior single-episode MDD diagnosis. Those MDD diagnoses are identified by the following ICD-9-CM/ICD-10-CM codes: 296.21/F32.0 (single episode, mild), 296.22/F32.1 (single episode, moderate), or 296.23/F32.2 (single episode, severe without mention of psychotic behavior). Common depression ICD-9-CM/ICD-10-CM codes for individuals that were not MDD diagnoses considered in this study included depressive disorder not elsewhere classified (311), dysthymic disorder (300.4/F43.1), and recurrent depression (296.3X/F33.X). These other depression diagnoses were excluded as their treatments and risk of disability would be different than the MDD diagnoses focused on in this study. To ensure that only patients diagnosed with depression in the health plans reporting to the MarketScan® consortium were included, patients who filled an antidepressant prescription in the 180 days prior to their first depression diagnosis and those whose first reported antidepressant was a refill prescription were also excluded.

### Pharmacological and psychotherapy treatments

Antidepressants were identified by matching the pharmaceutical claim’s National Drug Code (NDC) to NDCs identified as antidepressants by the therapeutic class in IBM’s Micromedex® RED BOOK®. Psychiatric visits were defined using the CCAE’s procedure group field and included psychotherapy (individual, family, or group), psychiatric advice, and therapeutic psychiatric services.

### Treatment adherence

The proportion of days covered (PDC) in the acute phase of treatment was used to measure a patient’s adherence to his or her antidepressant medication. The PDC has been used as a medication adherence metric by organizations such as the Centers for Disease Control and Prevention and the Pharmacy Quality Alliance [21]. Patients with a PDC ratio greater than or equal 0.80 were considered adherent, whereas patients filling an antidepressant prescription but with a PDC less than 0.80 were categorized as non-adherent.

Currently, depression treatment guidelines have not defined an optimal frequency of psychotherapy. However, the American Psychiatric Association (APA) and the Canadian Network for Mood and Anxiety Treatments (CANMAT) guidelines state that more intensive psychotherapy at the beginning of therapy may help obtain treatment goals [22, 23]. Absent of a set psychotherapy adherence metric, this research defined psychotherapy adherence in the acute phase as having at least four psychotherapy visits within the first 4 weeks after starting psychotherapy treatment [14]. Patients who had at least one psychotherapy visit, but did not have four visits in the first 4 weeks of psychotherapy treatment were categorized as non-adherent.

### Patient demographics and comorbidities

The following demographic variables were abstracted from the CCAE database for each patient: age, sex, geographic location, job industry, the health plan at the time of first depression diagnosis, salary/hourly status, and union status. The patient’s metropolitan statistical area was mapped to a U.S. Census Bureau estimate of rurality to determine if a patient was from a rural or urban geographic area [24].

Comorbidities documented during the year prior to the first depression diagnosis were defined using diagnosis groupings from Quan et al. [25] (e.g., diabetes, hypertension), with the exception of groups related to mental health conditions such as alcohol abuse, drug abuse, and psychoses. To gain more refinement in these mental health comorbidities, the multi-level CCS groups in the “mental illness” category were utilized. Given that MDD treatment may differ for women with a recent pregnancy and the risk of a work leave may increase due to complications from birth, pregnancy diagnoses and work leaves in the year before and after the first depression diagnosis were flagged using the “Complications of pregnancy; childbirth; and the puerperium” or “Certain conditions originating in the perinatal period” single-level CCS groupers.

### Outcome and follow-up time

The primary outcome was a patient’s first work leave, not related to a pregnancy, following their depression diagnosis. Work leaves in the HPM database are disability claims defined as short-term disability, long-term disability, or workers’ compensation claims. The reason for disability, captured as an ICD-9-CM or ICD-10-CM code in the HPM database, was missing in 1.7% of disability claims. Missing diagnoses for workers’ compensation claims were randomly imputed using the empirical distribution of workers’ compensation claims with known diagnoses. For non-occupational disability, missing diagnoses were imputed by first sub-setting the disability claims with a known reason for disability by sex, then randomly imputing the unknown diagnosis using the sex-stratified empirical distribution.

Follow-up time ended with 1) the patient’s first reported work leave following their depression diagnosis, 2) the end of disability benefits eligibility, 3) the end of health insurance eligibility, or 4) 2 years of elapsed time from the depression diagnosis. Follow-up time was censored at 2 years under the assumption that treatment in the acute phase was unlikely to influence work leave after that length of time. Time on a pregnancy-related work leave (*n* = 64, 0.2% of study population) was subtracted from the follow-up time, as individuals on a pregnancy leave are not “at risk” for another work leave. Pregnancy claims were flagged using the single-level CCS groupers previously described.

### Statistical analyses

Statistical analyses were performed using the R statistical computing software, Version 3.6.1 [26]. Chi-squared tests were used to test the difference in characteristics between 1) patients with a work leave in the acute phase, 2) patients with a work leave after the acute phase, and 3) patients without a work leave in the follow-up period. When evaluating the influence of antidepressant and psychotherapy adherence on future work leaves, patients were excluded if they had a non-pregnancy work leave in the acute phase of treatment (*n* = 5600, 21.3%), with the assumption that treatment did not have enough time to influence a patient’s work leave outcome. Only 712 patients of the 20,656 patients (3.4%) did not receive treatment in the form of either antidepressants or psychotherapy in the acute phase, making this comparison group statistically too small to compare to patients receiving treatment. Therefore, these 712 patients were dropped for both univariate and multiple variable analysis testing the influence of adherence to antidepressants and psychotherapy.

In univariate analysis, non-parametric Kaplan-Meier (KM) estimates were used to test the influence of antidepressant or psychotherapy treatment with time to a non-pregnancy work leave for the following adherence groups: 1) not treated with specified treatment (antidepressant or psychotherapy), 2) non-adherent to specified treatment, and 3) adherent patients to specified treatment. A KM estimate was also used to test the influence of adherence to either antidepressant or psychotherapy on future work leaves compared to patients not adherent to both treatments. Chi-squared p-values from log-rank tests were used to assess the statistical significance between the groups. To visually inspect the differences in work leave rates by treatment groups, Kaplan-Meier curves were generated using the *survminer* package [27].

Next, five multiple variable Cox proportional hazards models were used to test the difference in work leave risk by treatment adherence, while controlling for covariates. The models compared the following groups: 1) patients non-adherent to either antidepressant or psychotherapy treatment versus patients adherent to either antidepressant or psychotherapy treatment; 2) patients non-adherent and adherent to antidepressant treatment versus patients without antidepressant treatment; 3) patients adherent to antidepressant treatment versus patients non-adherent to antidepressant treatment; 4) patients non-adherent and adherent to psychotherapy treatment versus patients without psychotherapy treatment; and 5) patients adherent to psychotherapy treatment versus patients non-adherent to psychotherapy treatment.

All models adjusted for depression severity, history of a previous non-pregnancy work leave in year prior, age, sex, union membership, rurality of patient, industry of patient’s employer, health plan enrolled at time of depression diagnosis, comorbidities, and whether the patient was pregnant in the year prior to or after the depression diagnosis. Covariates were selected if available in the claims database and previous research has found an association with the exposure (psychotherapy or antidepressant treatment) or outcome (disability) [14, 28–30]. Models 2–5 adjusted for the alternative treatment (i.e., models focusing on antidepressant treatment controlled for psychotherapy treatment). Information about whether the employee was salaried was missing in 19.1% of cases and was not used in the multiple variable analysis. Other variables with missing values were imputed using the observed empirical distribution, with a maximum missingness of 6.6%. For the industry and health plan variables, categories including less than 10% of the total population were combined into a single “other” category.

To assess the influence of imputing the leave diagnosis for patients with a missing reason for work leave, the Cox proportional hazards models were re-run after dropping these patients with an imputed leave diagnosis. The significance of the hazard ratios produced by the Cox models were assessed with Wald’s test statistics from partial log-likelihood estimates. Ties were handled with the Efron method, the default method for R’s *survival* package [31].

## Results

A total of 26,256 patients were included in the analysis (Table 1). There were slightly more female (55.3%) than male patients, with an average age was 40 years old (standard deviation = 10 years). The majority of patients were non-union employees (70.7%), from a non-rural location (91.1%), and enrolled in a preferred provider organization (PPO) health plan at the time of their depression diagnosis (58.2%). About half of patients were diagnosed as having moderate MDD (50.5%), compared to mild (21.4%) or severe depression (28.1%). The majority of the study population were diagnosed in the outpatient setting (98.9%).

**Table 1:** Demographics of study populations, number in category (%)

Categories	All, N = 26,256
**Initial diagnosis**	
Mild depression	5618 (21.4%)
Moderate depression	13,259 (50.5%)
Severe depression without psychotic behavior	7379 (28.1%)
**Sex**	
Female	14,532 (55.3%)
Male	11,724 (44.7%)
**Industry**	
Construction	14 (0.1%)
Finance, insurance, real estate	7039 (26.8%)
Manufacturing, durable goods	5393 (20.5%)
Manufacturing, nondurable goods	2224 (8.5%)
Oil & gas extraction, mining	82 (0.3%)
Retail trade	1211 (4.6%)
Services	3665 (14.0%)
Transportation, communications, utilities	6460 (24.6%)
Wholesale	81 (0.3%)
Missing	87 (0.3%)
**Salaried**	
No	11,083 (42.2%)
Yes	10,154 (38.7%)
Missing	5019 (19.1%)
**Union**	
No	18,551 (70.7%)
Yes	5973 (22.7%)
Missing	1732 (6.6%)
**Health plan type at time of depression diagnosis**	
Consumer-driven health plan	2658 (10.1%)
Comprehensive	637 (2.4%)
Exclusive provider organization	294 (1.1%)
High-deductible health plan	1412 (5.4%)
Health maintenance organization	3030 (11.5%)
Point-of-service plan	2761 (10.5%)
Preferred provider organization	15,279 (58.2%)
Missing	185 (0.7%)
**Rural location of employee**	
No	23,926 (91.1%)
Yes	2330 (8.9%)

Of the 26,256 patients, 5600 patients (21.3%) had a non-pregnancy work leave (herein “work leave”) in the acute phase of treatment, 2300 patients (8.8%) had a work leave after the acute phase, and 18,356 patients (69.9%) did not have a work leave in the follow-up period (Supplemental Material Table S1). The most common type of work leave occurring in the acute phase and after the acute phase was a short-term disability leave (95.3 and 86.0%, respectively).

Patients experiencing a work leave during the acute phase were more likely to experience a work leave related to a mental illness or depression than patients experiencing a work leave after the acute phase (*p*-values < 0.0001, Table S1). Further, the depression severity was higher for patients experiencing a work leave in the acute phase (*p*-value < 0.0001). Potentially due to their increased depression severity, patients with a work leave in the acute phase were also more likely to be adherent to antidepressant or psychotherapy treatment (51.2%) than patients with a work leave after the acute phase (39.4%) or patients with no work leave (44.9%)(Table 2, *p*-values < 0.0001).

**Table 2:** Counts (%) of patients receiving antidepressant and psychotherapy treatment by population subset. Work leaves do not include pregnancy-related work leaves

Antidepressant treatment	Psychotherapy treatment	Work leave in acute phase, ***n*** = 5600	Work leave after acute phase, ***n*** = 2300	No work leave in follow-up period, ***n*** = 18,356
No	No	211 (3.8%)	81 (3.5%)	629 (3.4%)
No	Non-adherent	852 (15.2%)	643 (28%)	5043 (27.5%)
No	Adherent	772 (13.8%)	338 (14.7%)	3688 (20.1%)
Non-adherent	No	572 (10.2%)	365 (15.9%)	2440 (13.3%)
Non-adherent	Non-adherent	1100 (19.6%)	304 (13.2%)	2001 (10.9%)
Non-adherent	Adherent	1180 (21.1%)	160 (7%)	1297 (7.1%)
Adherent	No	213 (3.8%)	205 (8.9%)	1560 (8.5%)
Adherent	Non-adherent	265 (4.7%)	130 (5.7%)	1030 (5.6%)
Adherent	Adherent	435 (7.8%)	74 (3.2%)	668 (3.6%)

**Fig. 2:**
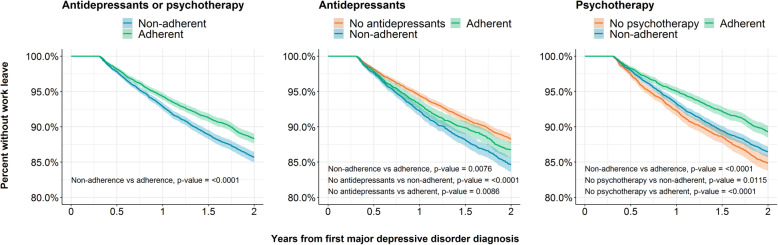
Kaplan-Meier curves assessing the association between antidepressant and psychotherapy adherence with time to a non-pregnancy work leave. *P*-values are calculated from chi-squared log rank tests between treatment groups

In univariate analyses, patients adherent to antidepressants and/or psychotherapy in the acute phase were less likely to have a work leave after the acute phase than non-adherent patients (*p*-value < 0.001). Patients with no antidepressant treatment were less likely to have a work leave compared to patients treated with antidepressants, whether adherent (*p*-value = 0.0045) or non-adherent (*p*-value < 0.0001, Fig. 2). Of patients prescribed with antidepressant treatment, adherent patients were less likely to have a work leave than non-adherent patients (*p*-value = 0.0076, Fig. 2). Patients adherent to psychotherapy treatment were less likely to have a work leave than patients with no psychotherapy treatment or those non-adherent to psychotherapy (*p*-values < 0.0001). The univariate relationships between treatment and work leaves were generally consistent when the population was subset by depression severity (Figure S1).

In the multiple variable Cox proportional hazards models, patients adherent to either antidepressants or psychotherapy in the acute phase had a 16% risk reduction in experiencing a work leave after the acute phase compared to patients not adherent to either antidepressant or psychotherapy treatments (HR = 0.84, 95% CI = 0.77–0.91). Patients who were non-adherent or adherent to antidepressant treatment had a 22% (HR = 1.22, 95% CI = 1.11–1.35) and 13% (HR = 1.13, 95% CI = 1.01–1.27) greater risk of a future work leave, respectively, than patients not receiving antidepressant treatment (Table 3). When comparing within the antidepressant treatment group, there was no significant difference in risk of a future work leave between adherent and non-adherent patients (*p*-value > 0.05). Conversely, patients receiving any psychotherapy treatment had a reduced risk of a future work leave. For example, patients who were non-adherent or adherent to psychotherapy treatment had a 9% (HR = 0.91, 95% CI = 0.81–1.02) and 28% (HR = 0.72, 95% CI = 0.64–0.82) reduced risk of a future work leave, respectively, than patients not receiving psychotherapy treatment (Table 3). When comparing within the psychotherapy treatment group, patients who were adherent to psychotherapy were less likely to have a work leave than non-adherent patients (HR = 0.80, 95% CI = 0.73–0.89). Sensitivity analyses excluding patients with missing diagnoses for their work leave did not change the findings (Table S7).

**Table 3:** Results from multiple variable Cox proportional hazards models testing the influence of antidepressant and psychotherapy treatment on work leaves. Full model results including covariates presented in Supporting Information Tables S2-S6

Reference Group	Treatment Group (no = 0, yes = 1)	Hazard Ratio	95% Confidence Interval	***p***-value
Non-adherent to both antidepressant and psychotherapy treatment^a^	Adherent to either antidepressant or psychotherapy treatment^a^	0.84	0.77–0.91	0.0001
No antidepressant treatment	Non-adherent to antidepressant treatment	1.22	1.11–1.35	0.0001
	Adherent to antidepressant treatment	1.13	1.01–1.27	0.0273
Non-adherent to antidepressant treatment^b^	Adherent to antidepressant treatment^b^	0.92	0.82–1.03	0.1439
No psychotherapy treatment	Non-adherent to psychotherapy treatment	0.91	0.81–1.02	0.0997
	Adherent to psychotherapy treatment	0.72	0.64–0.82	< 0.0001
Non-adherent to psychotherapy treatment^c^	Adherent to psychotherapy treatment^c^	0.80	0.73–0.89	< 0.0001

^a^ Analysis excludes patients without antidepressant or psychotherapy treatment

^b^ Analysis excludes patients without antidepressant treatment

^c^ Analysis excludes patients without psychotherapy treatment

## Discussion

In a population of 26,256 newly diagnosed patients with MDD, 21.3% had a work leave prior to the end of the acute phase of treatment. For individuals without a work leave in the acute phase of treatment and received antidepressant and/or psychotherapy treatment, treatment adherence reduced the likelihood of a future work leave for patients with newly diagnosed MDD. Psychotherapy treatment was more effective than antidepressants in reducing the risk of a future work leave.

The majority of work leaves in the follow-up period occurred in the acute phase of treatment (71.2%). This finding suggests that the symptoms of MDD need to be detected earlier to allow time for a clinician to diagnose and treatment(s) to be administered, monitored, and modified if needed. Patients may be waiting until they experience loss of functioning before seeking treatment. Increased depression screening, stigma reduction programs, and mental health resource outreach may be warranted for earlier detection [32]. Future research should consider how earlier detection and diagnosis of depression may be accomplished in a working population and examine how this may help reduce future work leaves.

The results of this research neither support nor contradict the findings from Burton et al. (2007) who observed that antidepressant treatment adherence reduced the probability of work leave compared to patients who were non-adherent to antidepressant treatment. In this study, patients who were adherent to antidepressant treatment did have an 8% risk reduction in future work leaves when compared to patients who were non-adherent to antidepressant treatment, but this relationship was not significant (*p*-value > 0.05).

The most important finding from this research is that psychotherapy treatment was more effective at reducing the likelihood of a future work leaves than antidepressant treatment. These findings are in line with previous research indicating that psychotherapy is more efficacious at improving quality of life compared to pharmacotherapy [10]. Whereas antidepressants treat the symptoms of depression, psychotherapy addresses the causal mechanisms including negative ideation and self-defeating behaviors patterns. As a results, psychotherapy has been shown to have a more enduring effect than antidepressants [33], potentially reducing the risk of relapse or recurrence in our study population. Antidepressants have also been shown to be ineffective in mild depression [34], while psychotherapy has been shown to be effective across the severity spectrum [35]. Although depression severity was controlled for in our survival models, the differential effectiveness may have persisted given depression severity was ascertained from medical claim diagnoses that are prone to misclassification.

Of note, the psychotherapy adherence metric used in this study was previously developed as a research initiative by Gaspar et al. 2019 because there is no set adherence metric for psychotherapy frequency by either the APA or CANMAT. Future research should validate the utility of this metric for predicting future work leaves and other functional capacity or quality of life outcomes.

Employers may want to ensure that the health care benefits they offer to their employees adequately cover the cost of psychotherapy visits for patients diagnosed with depression to incentivize the use of these services. In addition, employers may want to offer a flexible work schedule to allow the employee to utilize the treatment. Although offering flexible work schedules to allow employees to receive psychotherapy may cause intermittent lost work time, this short-term cost may be surpassed by the long-term benefit of improved productivity and reduced number of disability episodes.

The strengths of this research include using a national data sample including a large population of patients with MDD, increasing statistical power to detect associations. The MarketScan® databases cover many important confounders including comorbidities, past work leaves, sex, and work industry. Limitations of our research include using a retrospective claims analysis in defining a study population of newly diagnosed MDD patients. Previous research has shown that most algorithms selecting patients with depression from administrative data have shortcomings [20]. In addition, depression severity was defined using the medical code diagnosis and not depression severity scores, which may be more accurate. This study also used an indirect method of assessing antidepressant adherence, whereas direct methods including direct observations or antidepressant biomonitoring would represent a more accurate approach to determine adherence [36]. Determining the type of psychotherapy administered is problematic in claims data; therefore, this study grouped all psychotherapy together into one treatment group, although previous research has shown differential effects on outcomes by types of psychotherapy. For example, cognitive behavior therapy is a common psychotherapy technique that has been shown to be more effective than other therapies [37]. Some unmeasured confounding patient-level characteristics were not available in our dataset, such as education [38], race [39], and socioeconomic status [40]. This study did not account for differences in criteria for disability leave among the plans and organizations including elimination period. Finally, although health plan type was controlled for in our models, information regarding the number and frequency of covered psychotherapies within the health plans was unknown.

## Conclusion

Increased depression screening or mental health resource outreach may be warranted given that the majority of MDD patients with a work leave experienced their leave during the acute phase of treatment. Treatment adherence may reduce the likelihood of a future work leave for patients with newly diagnosed MDD. Given psychotherapy treatment appears more effective than antidepressants in reducing the risk of a future work leave, clinicians and employers should work together to provide access to psychotherapy and promote the benefit of adherence to psychotherapy treatment.

## Supplementary information

**Additional file 1.**

## Data Availability

Study data were licensed from a third party, International Business Machines (IBM) Watson Health, which prohibits the authors from making the data sets publicly available. Interested researchers may contact IBM Watson Health (ibm.com/watsonhealth) to apply to gain access to the Commercial Claims and Encounters and the Health and Productivity Management research databases in the same way the authors obtained the data. Any analysis, interpretation, or conclusion based on these data is solely that of the authors and not IBM Corporation.

## Abbreviations

APA: American Psychiatric Association
CANMAT: Canadian Network for Mood and Anxiety Treatments
CCAE: Commercial Claims and Encounters
CCS: Clinical Classification
HPM: Health and Productivity Management
ICD-9-CM: International Classification of Diseases, Ninth Revision, Clinical Modification
ICD-10-CM: International Classification of Diseases, Tenth Revision, Clinical Modification
KM: Kaplan-Meier
MDD: Major depressive disorder
NDC: National Drug Code
PDC: Proportion of days covered
U.S.: United States

## References

[1] Rank MR, Hirschl TA (2014). The risk of developing a work disability across the adulthood years. Disabil Health J.

[2] Helgesson M, Johansson B, Nordqvist T, Lundberg I, Vingård E (2015). Sickness absence at a young age and later sickness absence, disability pension, death, unemployment and income in native swedes and immigrants. Eur J Pub Health.

[3] Gifford B (2017). Temporarily disabled workers account for a disproportionate share of health care payments. Health Aff.

[4] Rehm J, Shield KD (2019). Global burden of disease and the impact of mental and addictive disorders. Curr Psychiatry Rep.

[5] Zaidel CS, Ethiraj RK, Berenji M, Gaspar FW (2018). Health care expenditures and length of disability across medical conditions. J Occup Environ Med.

[6] Airaksinen J, Jokela M, Virtanen M, Oksanen T, Pentti J, Vahtera J (2017). Development and validation of a risk prediction model for work disability: multicohort study. Sci Rep.

[7] Birnbaum HG, Kessler RC, Kelley D, Ben-Hamadi R, Joish VN, Greenberg PE (2010). Employer burden of mild, moderate, and severe major depressive disorder: mental health services utilization and costs, and work performance. Depress Anxiety.

[8] Substance Abuse and Mental Health Services Administration. Key substance use and mental health indicators in the US: results from the 2018 National Survey on drug use and health. Rockville; 2019. Available from: https://www.samhsa.gov/data/.

[9] Cipriani A, Furukawa TA, Salanti G, Chaimani A, Atkinson LZ, Ogawa Y (2018). Comparative efficacy and acceptability of 21 antidepressant drugs for the acute treatment of adults with major depressive disorder: a systematic review and network meta-analysis. Lancet.

[10] Kamenov K, Twomey C, Cabello M, Prina AM, Ayuso-Mateos JL (2017). The efficacy of psychotherapy, pharmacotherapy and their combination on functioning and quality of life in depression: a meta-analysis. Psychol Med.

[11] Oluboka OJ, Katzman MA, Habert J, McIntosh D, MacQueen GM, Milev RV (2018). Functional recovery in major depressive disorder: providing early optimal treatment for the individual patient. Int J Neuropsychopharmacol.

[12] National Institute of Mental Health (2019). Major Depression.

[13] Keyloun KR, Hansen RN, Hepp Z, Gillard P, Thase ME, Devine EB (2017). Adherence and persistence across antidepressant therapeutic classes: a retrospective claims analysis among insured US patients with major depressive disorder (MDD). CNS Drugs.

[14] Gaspar FW, Zaidel CS, Dewa CS (2019). Rates and determinants of use of pharmacotherapy and psychotherapy by patients with major depressive disorder. Psychiatr Serv.

[15] Burton WN, Chen C, Conti DJ, Schultz AB, Edington DW (2007). The association of antidepressant medication adherence with employee disability absences. Am J Manag Care.

[16] IBM Corporation (2018). IBM marketscan databases for health services researchers.

[17] Department of Health & Human Services (2000). Protection of Human Subjects.

[18] Agency for Healthcare Research and Quality. Clinical Classifications Software for ICD-9 Diagnoses: Healthcare Cost and Utilization Project; 2016. [cited 2017 Aug 2]. Available from: http://www.ahrq.gov/data/hcup/.

[19] National Committee for Quality Assurance. HEDIS 2015: Healthcare Effectiveness Data and Information Set, vol. 1. Washington, DC; 2014..

[20] Townsend L, Walkup JT, Crystal S, Olfson M (2012). A systematic review of validated methods for identifying depression using administrative data. Pharmacoepidemiol Drug Saf.

[21] National Center for Chronic Disease Prevention and Health Promotion. Calculating proportion of days covered (PDC) for antihypertensive and antidiabetic medications: an evaluation guide for grantees. Center Dis Control. 2015; Available from: https://www.cdc.gov/dhdsp/docs/Med-Adherence-Evaluation-Tool.pdf.

[22] American Psychiatric Association (2010). Practice guideline for the treatment of patients with major depressive disorder.

[23] Parikh SV, Quilty LC, Ravitz P, Rosenbluth M, Pavlova B, Grigoriadis S (2016). Canadian network for mood and anxiety treatments (CANMAT) 2016 clinical guidelines for the management of adults with major depressive disorder: section 2. Psychological treatments. Can J Psychiatry.

[24] United States Census (2010). County Rurality Level.

[25] Quan H, Sundararajan V, Halfon P, Fong A, Burnand B, Luthi JC (2005). et al. Coding algorithms for defining comorbidities in ICD-9-CM and ICD-10 administrative data. Med Care.

[26] R Core Team. R: a language and environment for satistical computing. Vienna; 2019. Available from: https://www.r-project.org/.

[27] Kassambara A, Kosinski M (2019). Survminer: Drawing Survival Curves using “ggplot2”.

[28] Gaspar FW, Zaidel CS, Dewa CS (2018). Rates and predictors of recurrent work disability due to common mental health disorders in the United States. PLoS One.

[29] de Vries H, Fishta A, Weikert B, Rodriguez Sanchez A, Wegewitz U (2018). Determinants of sickness absence and return to work among employees with common mental disorders: a scoping review. J Occup Rehabil.

[30] Dewa CS, Chau N, Dermer S (2009). Factors associated with short-term disability episodes. J Occup Environ Med.

[31] Therneau TM (2015). A Package for Survival Analysis in S.

[32] Dewa CS (2014). Worker attitudes towards mental health problems and disclosure. Int J Occup Environ Med.

[33] DeRubeis RJ, Siegle GJ, Hollon SD (2008). Cognitive therapy vs. medications for depression: treatment outcomes and neural mechanisms. Nat Rev Neurosci.

[34] Kirsch I, Deacon BJ, Huedo-Medina TB, Scoboria A, Moore TJ, Johnson BT (2008). Initial severity and antidepressant benefits: a meta-analysis of data submitted to the food and drug administration. PLoS Med.

[35] Cuijpers P, Smit F, Van Straten A (2007). Psychological treatments of subthreshold depression: a meta-analytic review. Acta Psychiatr Scand.

[36] Osterberg L, Blaschke T (2005). Adherence to medication. N Engl J Med.

[37] Tolin DF (2010). Is cognitive-behavioral therapy more effective than other therapies?. A meta-analytic review. Clin Psychol Rev.

[38] Martin-Vazquez M-J, Garcia-Toro M, Campoamor F, Pareja A, Aguirre I, Salva J (2011). Use and results of antidepressant treatment: patients’ perception. Curr Drug Ther.

[39] Rossom RC, Shortreed S, Coleman KJ, Beck A, Waitzfelder BE, Stewart C (2016). Antidepressant adherence across diverse populations and healthcare settings. Depress Anxiety.

[40] Jirón M, Escobar L, Arriagada L, Orellana S, Castro A (2011). Factores Asociados al Incumplimiento de los Tratamientos con Antidepresivos en Santiago, Chile. Value Health.

