# Supplementary material for: The influence of antidepressant and psychotherapy treatment adherence on future work leaves for patients with major depressive disorder
Source: BMC Psychiatry. 2020 Jun 19;20:320. doi: 10.1186/s12888-020-02731-9 (PMC7304154; doi:10.1186/s12888-020-02731-9)
Supplement: Supplementary file 1 — Additional file 1. [file 12888_2020_2731_MOESM1_ESM.docx]

**Supplemental Material**

1. **Table S1. Comparison of demographic information between patients with and without work leaves. Work leaves do not include pregnancy-related work leaves.**

| 1. **Categories** | 1. **Work leave in acute phase,** 2. **n = 5,600** | 1. **Work leave after acute phase,** 2. **n = 2,300** | 1. **No work leave in follow-up period,** 2. **n = 18,356** |
| --- | --- | --- | --- |
| 1. **Program type of work disability leave** |  |  |  |
| 1. Long-term disability | 1. 26 (0.5%) | 1. 14 (0.6%) | 1. 0 (0%) |
| 1. Short-term disability | 1. 5,334 (95.2%) | 1. 1,979 (86.0%) | 1. 0 (0%) |
| 1. Workers’ compensation | 1. 240 (4.3%) | 1. 307 (13.3%) | 1. 0 (0%) |
| 1. **Mental health work leave** |  |  |  |
| 1. No | 1. 1,191 (21.3%) | 1. 1,633 (71.0%) | 1. 18,356 (100.0%) |
| 1. Yes | 1. 4,409 (78.7%) | 1. 667 (29.0%) | 1. 0 (0%) |
| 1. **Depression work leave** |  |  |  |
| 1. No | 1. 2,264 (40.4%) | 1. 1,912 (83.1%) | 1. 18,356 (100.0%) |
| 1. Yes | 1. 3,336 (59.6%) | 1. 388 (16.9%) | 1. 0 (0%) |
| 1. **Initial diagnosis** |  |  |  |
| 1. Mild depression | 1. 578 (10.3%) | 1. 492 (21.4%) | 1. 4,548 (24.8%) |
| 1. Moderate depression | 1. 2,588 (46.2%) | 1. 1,170 (50.9%) | 1. 9,501 (51.8%) |
| 1. Severe depression | 1. 2,434 (43.5%) | 1. 638 (27.7%) | 1. 4,307 (23.5%) |
| 1. **Sex** |  |  |  |
| 1. Female | 1. 3,572 (63.8%) | 1. 1,294 (56.3%) | 1. 9,666 (52.7%) |
| 1. Male | 1. 2,028 (36.2%) | 1. 1,006 (43.7%) | 1. 8,690 (47.3%) |
| 1. **Industry** |  |  |  |
| 1. Construction | 1. 1 (<0.1%) | 1. 1 (<0.1%) | 1. 12 (0.1%) |
| 1. Finance, insurance, real estate | 1. 1,775 (31.7%) | 1. 464 (20.2%) | 1. 4,800 (26.1%) |
| 1. Manufacturing, durable goods | 1. 1,125 (20.1%) | 1. 639 (27.8%) | 1. 3,629 (19.8%) |
| 1. Manufacturing, nondurable goods | 1. 359 (6.4%) | 1. 213 (9.3%) | 1. 1,652 (9.0%) |
| 1. Oil & gas extraction, mining | 1. 1 (<0.1%) | 1. 2 (0.1%) | 1. 79 (0.4%) |
| 1. Retail trade | 1. 128 (2.3%) | 1. 67 (2.9%) | 1. 1,016 (5.5%) |
| 1. Services | 1. 259 (4.6%) | 1. 209 (9.1%) | 1. 3,197 (17.4%) |
| 1. Transportation, communications, utilities | 1. 1,952 (34.9%) | 1. 705 (30.7%) | 1. 3,803 (20.7%) |
| 1. Wholesale | 1. 0 (0%) | 1. 0 (0%) | 1. 81 (0.4%) |
| 1. Missing | 1. 0 (0%) | 1. 0 (0%) | 1. 87 (0.5%) |
| 1. **Salaried** |  |  |  |
| 1. No | 1. 3,539 (63.2%) | 1. 1,400 (60.9%) | 1. 6,144 (33.5%) |
| 1. Yes | 1. 1,337 (23.9%) | 1. 626 (27.2%) | 1. 8,191 (44.6%) |
| 1. Missing | 1. 724 (12.9%) | 1. 274 (11.9%) | 1. 4,021 (21.9%) |
| 1. **Union** |  |  |  |
| 1. No | 1. 3,558 (63.5%) | 1. 1,406 (61.1%) | 1. 13,587 (74.0%) |
| 1. Yes | 1. 1,690 (30.2%) | 1. 818 (35.6%) | 1. 3,465 (18.9%) |
| 1. Missing | 1. 352 (6.3%) | 1. 76 (3.3%) | 1. 1,304 (7.1%) |
| 1. **Health plan type at time of depression diagnosis** |  |  |  |
| 1. Consumer-driven health plan | 1. 454 (8.1%) | 1. 235 (10.2%) | 1. 1,969 (10.7%) |
| 1. Comprehensive | 1. 219 (3.9%) | 1. 89 (3.9%) | 1. 329 (1.8%) |
| 1. Exclusive provider organization | 1. 54 (1.0%) | 1. 19 (0.8%) | 1. 221 (1.2%) |
| 1. High-deductible health plan | 1. 201 (3.6%) | 1. 75 (3.3%) | 1. 1,136 (6.2%) |
| 1. Health maintenance organization | 1. 556 (9.9%) | 1. 244 (10.6%) | 1. 2,230 (12.1%) |
| 1. Point-of-service plan | 1. 627 (11.2%) | 1. 298 (13.0%) | 1. 1,836 (10.0%) |
| 1. Preferred provider organization | 1. 3,467 (61.9%) | 1. 1,332 (57.9%) | 1. 10,480 (57.1%) |
| 1. Missing | 1. 22 (0.4%) | 1. 8 (0.3%) | 1. 155 (0.8%) |
| 1. **Rural location of employee** |  |  |  |
| 1. No | 1. 5,174 (92.4%) | 1. 2,090 (90.9%) | 1. 16,662 (90.8%) |
| 1. Yes | 1. 426 (7.6%) | 1. 210 (9.1%) | 1. 1,694 (9.2%) |

1. **Table S2. Full results from Cox proportional hazards models comparing risk of non-pregnancy work leave between the following groups: 1) not adherent to either antidepressant or psychotherapy as reference group and 2) adherent to either antidepressant or psychotherapy treatment. HR = hazard ratio. CI = Confidence Interval**

| 1. **Variable** | 1. **HR** | 1. **95% CI** | 1. **p-value** |
| --- | --- | --- | --- |
| 1. **Adherent to either antidepressant or psychotherapy treatment. Non-adherent to either antidepressant or psychotherapy as reference group (no = 0, yes = 1)** | 1. 0.84 | 1. 0.77-0.91 | 1. 0.0001 |
| 1. **Depression severity, mild as baseline (no = 0, yes = 1)** |  |  |  |
| 1. Moderate depression | 1. 1.11 | 1. 1.00-1.24 | 1. 0.0524 |
| 1. Severe depression | 1. 1.33 | 1. 1.17-1.50 | 1. <0.0001 |
| 1. **Non-pregnancy work leave in year prior to first depression diagnosis (no = 0, yes = 1)** | 1. 3.26 | 1. 2.91-3.66 | 1. <0.0001 |
| 1. **Age (years)** | 1. 1.00 | 1. 1.00-1.01 | 1. 0.4405 |
| 1. **Sex (female = 0, male = 1)** | 1. 0.75 | 1. 0.69-0.83 | 1. <0.0001 |
| 1. **Union (no = 0, yes = 1)** | 1. 2.06 | 1. 1.85-2.28 | 1. <0.0001 |
| 1. **Rural (no = 0, yes = 1)** | 1. 0.90 | 1. 0.78-1.05 | 1. 0.1827 |
| 1. **Employee industry. Baseline is patients in industries representing <10% of total population (no = 0, yes = 1)** |  |  |  |
| 1. Finance, Insurance, Real Estate | 1. 0.97 | 1. 0.83-1.14 | 1. 0.7397 |
| 1. Manufacturing, Durable Goods | 1. 1.30 | 1. 1.12-1.50 | 1. 0.0006 |
| 1. Services | 1. 0.48 | 1. 0.39-0.58 | 1. <0.0001 |
| 1. Transportation, Communications, Utilities | 1. 1.38 | 1. 1.19-1.60 | 1. <0.0001 |
| 1. **Employee health plan at time of depression diagnosis. Baseline is patients in health plans representing <10% of total population (no = 0, yes = 1)** |  |  |  |
| 1. Consumer-driven health plan | 1. 1.16 | 1. 0.95-1.42 | 1. 0.147 |
| 1. Health maintenance organization | 1. 1.08 | 1. 0.87-1.33 | 1. 0.4845 |
| 1. Point-of-service plan | 1. 0.97 | 1. 0.79-1.20 | 1. 0.7883 |
| 1. Preferred provider organization | 1. 1.16 | 1. 0.99-1.36 | 1. 0.0702 |
| 1. **Comorbidities noted in year prior to depression diagnosis (no = 0, yes = 1)** |  |  |  |
| 1. Adjustment disorders | 1. 0.87 | 1. 0.75-0.99 | 1. 0.0393 |
| 1. Deficiency anemia | 1. 1.26 | 1. 0.96-1.64 | 1. 0.0919 |
| 1. Anxiety disorders | 1. 1.10 | 1. 0.98-1.24 | 1. 0.119 |
| 1. Cardiac arrhythmias | 1. 1.01 | 1. 0.80-1.29 | 1. 0.9229 |
| 1. Attention deficit disorder and attention deficit hyperactivity disorder | 1. 1.03 | 1. 0.73-1.46 | 1. 0.8506 |
| 1. Bipolar disorders | 1. 1.23 | 1. 0.89-1.71 | 1. 0.2031 |
| 1. Codes related to substance-related disorders | 1. 1.15 | 1. 0.91-1.46 | 1. 0.2386 |
| 1. Diabetes mellitus | 1. 1.13 | 1. 0.95-1.35 | 1. 0.1635 |
| 1. Diabetes mellitus with complications | 1. 1.03 | 1. 0.73-1.46 | 1. 0.8607 |
| 1. Fluid and electrolyte disorders | 1. 1.02 | 1. 0.75-1.39 | 1. 0.9068 |
| 1. Hypertension | 1. 1.26 | 1. 1.12-1.41 | 1. 0.0001 |
| 1. Hypothyroidism | 1. 0.94 | 1. 0.79-1.12 | 1. 0.4878 |
| 1. Liver disease | 1. 1.21 | 1. 0.94-1.57 | 1. 0.1379 |
| 1. Other neurological disorders | 1. 1.23 | 1. 0.89-1.71 | 1. 0.205 |
| 1. Obesity | 1. 1.26 | 1. 1.08-1.49 | 1. 0.0045 |
| 1. Chronic pulmonary disease | 1. 1.20 | 1. 1.04-1.39 | 1. 0.0152 |
| 1. Rheumatoid arthritis/collagen vascular diseases | 1. 1.35 | 1. 1.04-1.76 | 1. 0.0239 |
| 1. Substance-related disorders | 1. 1.30 | 1. 0.96-1.78 | 1. 0.0931 |
| 1. Solid tumor without metastasis | 1. 1.02 | 1. 0.77-1.37 | 1. 0.87 |
| 1. Valvular disease | 1. 1.25 | 1. 0.94-1.68 | 1. 0.1299 |
| 1. **Pregnancy in year before depression diagnosis (no = 0, yes = 1)** | 1. 0.92 | 1. 0.72-1.19 | 1. 0.5434 |
| 1. **Pregnancy in year after depression diagnosis (no = 0, yes = 1)** | 1. 1.77 | 1. 1.40-2.24 | 1. <0.0001 |
| 1. **Year of depression diagnosis. Baseline is 2008. (no = 0, yes = 1)** |  |  |  |
| 1. 2009 | 1. 1.34 | 1. 1.08-1.66 | 1. 0.0085 |
| 1. 2010 | 1. 1.61 | 1. 1.31-1.99 | 1. <0.0001 |
| 1. 2011 | 1. 1.75 | 1. 1.42-2.16 | 1. <0.0001 |
| 1. 2012 | 1. 1.51 | 1. 1.22-1.88 | 1. 0.0002 |
| 1. 2013 | 1. 1.57 | 1. 1.25-1.96 | 1. 0.0001 |
| 1. 2014 | 1. 1.68 | 1. 1.35-2.10 | 1. <0.0001 |
| 1. 2015 | 1. 1.95 | 1. 1.57-2.42 | 1. <0.0001 |
| 1. 2016 | 1. 2.17 | 1. 1.75-2.68 | 1. <0.0001 |

1. **Table S3. Full results from Cox proportional hazards models comparing risk of non-pregnancy work leave between the following groups: 1) no antidepressant treatment, 2) non-adherent to antidepressant treatment, and 3) adherent to antidepressant treatment. HR = hazard ratio. CI = Confidence Interval**

| 1. **Variable** | 1. **HR** | 1. **95% CI** | 1. **p-value** |
| --- | --- | --- | --- |
| 1. **Antidepressant treatment. No antidepressant treatment as reference group (no = 0, yes = 1)** |  |  |  |
| 1. Non-adherent to antidepressant treatment | 1. 1.22 | 1. 1.11-1.35 | 1. 0.0001 |
| 1. Adherent to antidepressant treatment | 1. 1.13 | 1. 1.01-1.27 | 1. 0.0273 |
| 1. **Adherent to psychotherapy treatment. Not adherent to psychotherapy treatment as reference group (no = 0, yes = 1)** | 1. 0.80 | 1. 0.72-0.88 | 1. <0.0001 |
| 1. **Depression severity, mild as baseline (no = 0, yes = 1)** |  |  |  |
| 1. Moderate depression | 1. 1.10 | 1. 0.99-1.23 | 1. 0.0852 |
| 1. Severe depression | 1. 1.31 | 1. 1.16-1.48 | 1. <0.0001 |
| 1. **Non-pregnancy work leave in year prior to first depression diagnosis (no = 0, yes = 1)** | 1. 3.27 | 1. 2.91-3.67 | 1. <0.0001 |
| 1. **Age (years)** | 1. 1.00 | 1. 1.00-1.01 | 1. 0.4733 |
| 1. **Sex (female = 0, male = 1)** | 1. 0.76 | 1. 0.69-0.83 | 1. <0.0001 |
| 1. **Union (no = 0, yes = 1)** | 1. 2.08 | 1. 1.87-2.30 | 1. <0.0001 |
| 1. **Rural (no = 0, yes = 1)** | 1. 0.90 | 1. 0.78-1.05 | 1. 0.1819 |
| 1. **Employee industry. Baseline is patients in industries representing <10% of total population (no = 0, yes = 1)** |  |  |  |
| 1. Finance, Insurance, Real Estate | 1. 1.00 | 1. 0.85-1.16 | 1. 0.9747 |
| 1. Manufacturing, Durable Goods | 1. 1.32 | 1. 1.13-1.53 | 1. 0.0003 |
| 1. Services | 1. 0.48 | 1. 0.39-0.59 | 1. <0.0001 |
| 1. Transportation, Communications, Utilities | 1. 1.41 | 1. 1.21-1.63 | 1. <0.0001 |
| 1. **Employee health plan at time of depression diagnosis. Baseline is patients in health plans representing <10% of total population (no = 0, yes = 1)** |  |  |  |
| 1. Consumer-driven health plan | 1. 1.17 | 1. 0.96-1.43 | 1. 0.1304 |
| 1. Health maintenance organization | 1. 1.09 | 1. 0.88-1.34 | 1. 0.4366 |
| 1. Point-of-service plan | 1. 0.97 | 1. 0.79-1.19 | 1. 0.7575 |
| 1. Preferred provider organization | 1. 1.16 | 1. 0.99-1.36 | 1. 0.0704 |
| 1. **Comorbidities noted in year prior to depression diagnosis (no = 0, yes = 1)** |  |  |  |
| 1. Adjustment disorders | 1. 0.89 | 1. 0.77-1.02 | 1. 0.0931 |
| 1. Deficiency anemia | 1. 1.25 | 1. 0.96-1.64 | 1. 0.0994 |
| 1. Anxiety disorders | 1. 1.11 | 1. 0.98-1.25 | 1. 0.0941 |
| 1. Cardiac arrhythmias | 1. 1.00 | 1. 0.79-1.28 | 1. 0.9685 |
| 1. Attention deficit disorder and attention deficit hyperactivity disorder | 1. 1.03 | 1. 0.73-1.45 | 1. 0.8798 |
| 1. Bipolar disorders | 1. 1.26 | 1. 0.91-1.74 | 1. 0.1642 |
| 1. Codes related to substance-related disorders | 1. 1.11 | 1. 0.88-1.41 | 1. 0.3693 |
| 1. Diabetes mellitus | 1. 1.13 | 1. 0.95-1.35 | 1. 0.1786 |
| 1. Diabetes mellitus with complications | 1. 1.01 | 1. 0.71-1.43 | 1. 0.9735 |
| 1. Fluid and electrolyte disorders | 1. 1.01 | 1. 0.74-1.38 | 1. 0.9515 |
| 1. Hypertension | 1. 1.24 | 1. 1.11-1.39 | 1. 0.0002 |
| 1. Hypothyroidism | 1. 0.93 | 1. 0.78-1.11 | 1. 0.4372 |
| 1. Liver disease | 1. 1.22 | 1. 0.95-1.58 | 1. 0.1238 |
| 1. Other neurological disorders | 1. 1.24 | 1. 0.89-1.71 | 1. 0.1976 |
| 1. Obesity | 1. 1.27 | 1. 1.08-1.49 | 1. 0.0043 |
| 1. Chronic pulmonary disease | 1. 1.20 | 1. 1.03-1.39 | 1. 0.0185 |
| 1. Rheumatoid arthritis/collagen vascular diseases | 1. 1.35 | 1. 1.04-1.76 | 1. 0.0229 |
| 1. Substance-related disorders | 1. 1.31 | 1. 0.96-1.78 | 1. 0.0876 |
| 1. Solid tumor without metastasis | 1. 1.02 | 1. 0.77-1.37 | 1. 0.869 |
| 1. Valvular disease | 1. 1.28 | 1. 0.95-1.71 | 1. 0.104 |
| 1. **Pregnancy in year before depression diagnosis (no = 0, yes = 1)** | 1. 0.92 | 1. 0.72-1.19 | 1. 0.5418 |
| 1. **Pregnancy in year after depression diagnosis (no = 0, yes = 1)** | 1. 1.82 | 1. 1.43-2.30 | 1. <0.0001 |
| 1. **Year of depression diagnosis. Baseline is 2008. (no = 0, yes = 1)** |  |  |  |
| 1. 2009 | 1. 1.34 | 1. 1.08-1.66 | 1. 0.008 |
| 1. 2010 | 1. 1.64 | 1. 1.33-2.01 | 1. <0.0001 |
| 1. 2011 | 1. 1.75 | 1. 1.42-2.15 | 1. <0.0001 |
| 1. 2012 | 1. 1.50 | 1. 1.21-1.87 | 1. 0.0002 |
| 1. 2013 | 1. 1.57 | 1. 1.25-1.96 | 1. 0.0001 |
| 1. 2014 | 1. 1.66 | 1. 1.33-2.07 | 1. <0.0001 |
| 1. 2015 | 1. 1.88 | 1. 1.52-2.34 | 1. <0.0001 |
| 1. 2016 | 1. 2.02 | 1. 1.63-2.50 | 1. <0.0001 |

1. **Table S4. Full results from Cox proportional-hazards models comparing risk of non-pregnancy work leave between the following groups: 1) non-adherent to antidepressant treatment and 2) adherent to antidepressant treatment. HR = hazard ratio. CI = Confidence Interval**

| 1. **Variable** | 1. **HR** | 1. **95% CI** | 1. **p-value** |
| --- | --- | --- | --- |
| 1. **Adherent to antidepressant treatment. Non-adherent to antidepressant treatment as reference group (no = 0, yes = 1)** | 1. 0.92 | 1. 0.82-1.03 | 1. 0.1439 |
| 1. **Psychotherapy treatment. No psychotherapy treatment as reference group (no = 0, yes = 1)** |  |  |  |
| 1. Non-adherent to psychotherapy treatment | 1. 0.96 | 1. 0.84-1.10 | 1. 0.565 |
| 1. Adherent to psychotherapy treatment | 1. 0.82 | 1. 0.69-0.96 | 1. 0.016 |
| 1. **Depression severity, mild as baseline (no = 0, yes = 1)** |  |  |  |
| 1. Moderate depression | 1. 1.09 | 1. 0.93-1.26 | 1. 0.2844 |
| 1. Severe depression | 1. 1.24 | 1. 1.04-1.47 | 1. 0.0143 |
| 1. **Non-pregnancy work leave in year prior to first depression diagnosis (no = 0, yes = 1)** | 1. 3.11 | 1. 2.65-3.64 | 1. <0.0001 |
| 1. **Age (years)** | 1. 1.01 | 1. 1.00-1.01 | 1. 0.0442 |
| 1. **Sex (female = 0, male = 1)** | 1. 0.78 | 1. 0.69-0.88 | 1. 0.0001 |
| 1. **Union (no = 0, yes = 1)** | 1. 2.01 | 1. 1.75-2.31 | 1. <0.0001 |
| 1. **Rural (no = 0, yes = 1)** | 1. 1.00 | 1. 0.83-1.20 | 1. 0.9674 |
| 1. **Employee industry. Baseline is patients in industries representing <10% of total population (no = 0, yes = 1)** |  |  |  |
| 1. Finance, Insurance, Real Estate | 1. 1.04 | 1. 0.85-1.28 | 1. 0.69 |
| 1. Manufacturing, Durable Goods | 1. 1.37 | 1. 1.13-1.67 | 1. 0.0013 |
| 1. Services | 1. 0.56 | 1. 0.44-0.72 | 1. <0.0001 |
| 1. Transportation, Communications, Utilities | 1. 1.46 | 1. 1.20-1.77 | 1. 0.0002 |
| 1. **Employee health plan at time of depression diagnosis. Baseline is patients in health plans representing <10% of total population (no = 0, yes = 1)** |  |  |  |
| 1. Consumer-driven health plan | 1. 1.22 | 1. 0.93-1.58 | 1. 0.1445 |
| 1. Health maintenance organization | 1. 1.08 | 1. 0.81-1.44 | 1. 0.588 |
| 1. Point-of-service plan | 1. 0.95 | 1. 0.72-1.26 | 1. 0.7192 |
| 1. Preferred provider organization | 1. 1.21 | 1. 0.98-1.49 | 1. 0.0796 |
| 1. **Comorbidities noted in year prior to depression diagnosis (no = 0, yes = 1)** |  |  |  |
| 1. Adjustment disorders | 1. 0.98 | 1. 0.81-1.19 | 1. 0.8716 |
| 1. Deficiency anemia | 1. 1.28 | 1. 0.91-1.80 | 1. 0.1634 |
| 1. Anxiety disorders | 1. 1.09 | 1. 0.93-1.28 | 1. 0.2908 |
| 1. Cardiac arrhythmias | 1. 1.04 | 1. 0.76-1.42 | 1. 0.7994 |
| 1. Attention deficit disorder and attention deficit hyperactivity disorder | 1. 1.11 | 1. 0.71-1.73 | 1. 0.6513 |
| 1. Bipolar disorders | 1. 1.21 | 1. 0.75-1.93 | 1. 0.4333 |
| 1. Codes related to substance-related disorders | 1. 1.23 | 1. 0.93-1.64 | 1. 0.1436 |
| 1. Diabetes mellitus | 1. 1.06 | 1. 0.84-1.34 | 1. 0.609 |
| 1. Diabetes mellitus with complications | 1. 1.13 | 1. 0.74-1.71 | 1. 0.575 |
| 1. Fluid and electrolyte disorders | 1. 1.01 | 1. 0.68-1.50 | 1. 0.943 |
| 1. Hypertension | 1. 1.22 | 1. 1.05-1.41 | 1. 0.0097 |
| 1. Hypothyroidism | 1. 1.01 | 1. 0.81-1.25 | 1. 0.9508 |
| 1. Liver disease | 1. 1.27 | 1. 0.91-1.77 | 1. 0.1646 |
| 1. Other neurological disorders | 1. 1.25 | 1. 0.82-1.92 | 1. 0.301 |
| 1. Obesity | 1. 1.18 | 1. 0.95-1.46 | 1. 0.1283 |
| 1. Chronic pulmonary disease | 1. 1.17 | 1. 0.96-1.43 | 1. 0.1116 |
| 1. Rheumatoid arthritis/collagen vascular diseases | 1. 1.35 | 1. 0.96-1.89 | 1. 0.0875 |
| 1. Substance-related disorders | 1. 1.51 | 1. 1.05-2.18 | 1. 0.0272 |
| 1. Solid tumor without metastasis | 1. 1.13 | 1. 0.79-1.63 | 1. 0.5014 |
| 1. Valvular disease | 1. 1.10 | 1. 0.71-1.70 | 1. 0.6743 |
| 1. **Pregnancy in year before depression diagnosis (no = 0, yes = 1)** | 1. 0.91 | 1. 0.65-1.29 | 1. 0.6095 |
| 1. **Pregnancy in year after depression diagnosis (no = 0, yes = 1)** | 1. 1.83 | 1. 1.28-2.62 | 1. 0.0009 |
| 1. **Year of depression diagnosis. Baseline is 2008. (no = 0, yes = 1)** |  |  |  |
| 1. 2009 | 1. 1.44 | 1. 1.06-1.95 | 1. 0.0201 |
| 1. 2010 | 1. 1.86 | 1. 1.38-2.50 | 1. <0.0001 |
| 1. 2011 | 1. 1.86 | 1. 1.39-2.49 | 1. <0.0001 |
| 1. 2012 | 1. 1.47 | 1. 1.08-2.00 | 1. 0.0156 |
| 1. 2013 | 1. 1.67 | 1. 1.22-2.29 | 1. 0.0013 |
| 1. 2014 | 1. 1.62 | 1. 1.19-2.21 | 1. 0.0023 |
| 1. 2015 | 1. 1.85 | 1. 1.37-2.50 | 1. 0.0001 |
| 1. 2016 | 1. 1.79 | 1. 1.33-2.41 | 1. 0.0001 |

1. **Table S5. Full results from Cox proportional hazards models comparing risk of non-pregnancy work leave between the following groups: 1) no psychotherapy treatment, 2) non-adherent to psychotherapy treatment, and 3) adherent to psychotherapy treatment. HR = hazard ratio. CI = Confidence Interval**

| 1. **Variable** | 1. **HR** | 1. **95% CI** | 1. **p-value** |
| --- | --- | --- | --- |
| 1. **Psychotherapy treatment. No psychotherapy treatment and non-adherent to psychotherapy as reference group (no = 0, yes = 1)** |  |  |  |
| 1. Non-adherent to psychotherapy treatment | 1. 0.91 | 1. 0.81-1.02 | 1. 0.0997 |
| 1. Adherent to psychotherapy treatment | 1. 0.72 | 1. 0.64-0.82 | 1. <0.0001 |
| 1. **Adherent to antidepressant treatment. Non-adherent to antidepressant treatment as reference group (no = 0, yes = 1)** | 1. 1.02 | 1. 0.91-1.13 | 1. 0.7723 |
| 1. **Depression severity, mild as baseline (no = 0, yes = 1)** |  |  |  |
| 1. Moderate depression | 1. 1.12 | 1. 1.00-1.25 | 1. 0.0454 |
| 1. Severe depression | 1. 1.35 | 1. 1.19-1.52 | 1. <0.0001 |
| 1. **Non-pregnancy work leave in year prior to first depression diagnosis (no = 0, yes = 1)** | 1. 3.26 | 1. 2.90-3.66 | 1. <0.0001 |
| 1. **Age (years)** | 1. 1.00 | 1. 1.00-1.01 | 1. 0.5418 |
| 1. **Sex (female = 0, male = 1)** | 1. 0.76 | 1. 0.69-0.83 | 1. <0.0001 |
| 1. **Union (no = 0, yes = 1)** | 1. 2.07 | 1. 1.87-2.30 | 1. <0.0001 |
| 1. **Rural (no = 0, yes = 1)** | 1. 0.91 | 1. 0.78-1.05 | 1. 0.2008 |
| 1. **Employee industry. Baseline is patients in industries representing <10% of total population (no = 0, yes = 1)** |  |  |  |
| 1. Finance, Insurance, Real Estate | 1. 0.99 | 1. 0.85-1.16 | 1. 0.9192 |
| 1. Manufacturing, Durable Goods | 1. 1.31 | 1. 1.13-1.52 | 1. 0.0004 |
| 1. Services | 1. 0.48 | 1. 0.39-0.58 | 1. <0.0001 |
| 1. Transportation, Communications, Utilities | 1. 1.40 | 1. 1.21-1.63 | 1. <0.0001 |
| 1. **Employee health plan at time of depression diagnosis. Baseline is patients in health plans representing <10% of total population (no = 0, yes = 1)** |  |  |  |
| 1. Consumer-driven health plan | 1. 1.16 | 1. 0.95-1.42 | 1. 0.1377 |
| 1. Health maintenance organization | 1. 1.08 | 1. 0.88-1.33 | 1. 0.4646 |
| 1. Point-of-service plan | 1. 0.97 | 1. 0.79-1.19 | 1. 0.7785 |
| 1. Preferred provider organization | 1. 1.16 | 1. 0.99-1.36 | 1. 0.0713 |
| 1. **Comorbidities noted in year prior to depression diagnosis (no = 0, yes = 1)** |  |  |  |
| 1. Adjustment disorders | 1. 0.89 | 1. 0.78-1.03 | 1. 0.1103 |
| 1. Deficiency anemia | 1. 1.26 | 1. 0.96-1.65 | 1. 0.0899 |
| 1. Anxiety disorders | 1. 1.12 | 1. 0.99-1.26 | 1. 0.0719 |
| 1. Cardiac arrhythmias | 1. 1.01 | 1. 0.80-1.29 | 1. 0.9199 |
| 1. Attention deficit disorder and attention deficit hyperactivity disorder | 1. 1.03 | 1. 0.73-1.46 | 1. 0.8577 |
| 1. Bipolar disorders | 1. 1.27 | 1. 0.92-1.75 | 1. 0.1511 |
| 1. Codes related to substance-related disorders | 1. 1.11 | 1. 0.88-1.41 | 1. 0.3687 |
| 1. Diabetes mellitus | 1. 1.13 | 1. 0.95-1.35 | 1. 0.1643 |
| 1. Diabetes mellitus with complications | 1. 1.00 | 1. 0.71-1.43 | 1. 0.9812 |
| 1. Fluid and electrolyte disorders | 1. 1.01 | 1. 0.74-1.38 | 1. 0.9502 |
| 1. Hypertension | 1. 1.25 | 1. 1.11-1.40 | 1. 0.0001 |
| 1. Hypothyroidism | 1. 0.94 | 1. 0.79-1.11 | 1. 0.4536 |
| 1. Liver disease | 1. 1.23 | 1. 0.95-1.58 | 1. 0.1187 |
| 1. Other neurological disorders | 1. 1.24 | 1. 0.89-1.71 | 1. 0.1976 |
| 1. Obesity | 1. 1.26 | 1. 1.07-1.48 | 1. 0.0049 |
| 1. Chronic pulmonary disease | 1. 1.20 | 1. 1.03-1.39 | 1. 0.0183 |
| 1. Rheumatoid arthritis/collagen vascular diseases | 1. 1.35 | 1. 1.04-1.76 | 1. 0.0231 |
| 1. Substance-related disorders | 1. 1.33 | 1. 0.98-1.81 | 1. 0.071 |
| 1. Solid tumor without metastasis | 1. 1.02 | 1. 0.76-1.36 | 1. 0.9153 |
| 1. Valvular disease | 1. 1.28 | 1. 0.95-1.71 | 1. 0.1037 |
| 1. **Pregnancy in year before depression diagnosis (no = 0, yes = 1)** | 1. 0.93 | 1. 0.72-1.20 | 1. 0.5913 |
| 1. **Pregnancy in year after depression diagnosis (no = 0, yes = 1)** | 1. 1.80 | 1. 1.42-2.28 | 1. <0.0001 |
| 1. **Year of depression diagnosis. Baseline is 2008. (no = 0, yes = 1)** |  |  |  |
| 1. 2009 | 1. 1.33 | 1. 1.07-1.65 | 1. 0.0093 |
| 1. 2010 | 1. 1.61 | 1. 1.31-1.99 | 1. <0.0001 |
| 1. 2011 | 1. 1.74 | 1. 1.41-2.14 | 1. <0.0001 |
| 1. 2012 | 1. 1.49 | 1. 1.20-1.86 | 1. 0.0003 |
| 1. 2013 | 1. 1.54 | 1. 1.24-1.93 | 1. 0.0001 |
| 1. 2014 | 1. 1.64 | 1. 1.31-2.04 | 1. <0.0001 |
| 1. 2015 | 1. 1.86 | 1. 1.50-2.31 | 1. <0.0001 |
| 1. 2016 | 1. 2.01 | 1. 1.62-2.49 | 1. <0.0001 |

1. **Table S6. Full results from Cox proportional hazards models comparing risk of non-pregnancy work leave between the following groups: 1) non-adherent to psychotherapy treatment and 2) adherent to psychotherapy treatment. HR = hazard ratio. CI = Confidence Interval**

| 1. **Variable** | 1. **HR** | 1. **95% CI** | 1. **p-value** |
| --- | --- | --- | --- |
| 1. **Adherent to psychotherapy treatment. Non-adherent to psychotherapy treatment as reference group (no = 0, yes = 1)** | 1. 0.80 | 1. 0.73-0.89 | 1. <0.0001 |
| 1. **Antidepressant treatment. No antidepressant treatment as reference group (no = 0, yes = 1)** |  |  |  |
| 1. Non-adherent to antidepressant treatment | 1. 1.22 | 1. 1.08-1.37 | 1. 0.0012 |
| 1. Adherent to antidepressant treatment | 1. 1.15 | 1. 1.00-1.32 | 1. 0.0536 |
| 1. **Depression severity, mild as baseline (no = 0, yes = 1)** |  |  |  |
| 1. Moderate depression | 1. 1.14 | 1. 0.99-1.30 | 1. 0.0615 |
| 1. Severe depression | 1. 1.39 | 1. 1.20-1.61 | 1. <0.0001 |
| 1. **Non-pregnancy work leave in year prior to first depression diagnosis (no = 0, yes = 1)** | 1. 3.44 | 1. 3.01-3.93 | 1. <0.0001 |
| 1. **Age (years)** | 1. 1.00 | 1. 0.99-1.00 | 1. 0.4033 |
| 1. **Sex (female = 0, male = 1)** | 1. 0.78 | 1. 0.70-0.87 | 1. <0.0001 |
| 1. **Union (no = 0, yes = 1)** | 1. 2.02 | 1. 1.79-2.27 | 1. <0.0001 |
| 1. **Rural (no = 0, yes = 1)** | 1. 0.86 | 1. 0.72-1.02 | 1. 0.0855 |
| 1. **Employee industry. Baseline is patients in industries representing <10% of total population (no = 0, yes = 1)** |  |  |  |
| 1. Finance, Insurance, Real Estate | 1. 0.99 | 1. 0.83-1.19 | 1. 0.9278 |
| 1. Manufacturing, Durable Goods | 1. 1.31 | 1. 1.10-1.57 | 1. 0.0027 |
| 1. Services | 1. 0.45 | 1. 0.35-0.57 | 1. <0.0001 |
| 1. Transportation, Communications, Utilities | 1. 1.39 | 1. 1.17-1.66 | 1. 0.0002 |
| 1. **Employee health plan at time of depression diagnosis. Baseline is patients in health plans representing <10% of total population (no = 0, yes = 1)** |  |  |  |
| 1. Consumer-driven health plan | 1. 1.15 | 1. 0.90-1.46 | 1. 0.2537 |
| 1. Health maintenance organization | 1. 1.10 | 1. 0.86-1.40 | 1. 0.4491 |
| 1. Point-of-service plan | 1. 1.05 | 1. 0.83-1.33 | 1. 0.6892 |
| 1. Preferred provider organization | 1. 1.12 | 1. 0.93-1.36 | 1. 0.2421 |
| 1. **Comorbidities noted in year prior to depression diagnosis (no = 0, yes = 1)** |  |  |  |
| 1. Adjustment disorders | 1. 0.85 | 1. 0.73-0.98 | 1. 0.0275 |
| 1. Deficiency anemia | 1. 1.47 | 1. 1.09-1.98 | 1. 0.012 |
| 1. Anxiety disorders | 1. 1.13 | 1. 0.99-1.29 | 1. 0.0805 |
| 1. Cardiac arrhythmias | 1. 1.02 | 1. 0.78-1.33 | 1. 0.8883 |
| 1. Attention deficit disorder and attention deficit hyperactivity disorder | 1. 1.06 | 1. 0.73-1.54 | 1. 0.769 |
| 1. Bipolar disorders | 1. 1.31 | 1. 0.94-1.82 | 1. 0.1171 |
| 1. Codes related to substance-related disorders | 1. 1.09 | 1. 0.80-1.48 | 1. 0.6064 |
| 1. Diabetes mellitus | 1. 1.15 | 1. 0.94-1.42 | 1. 0.1779 |
| 1. Diabetes mellitus with complications | 1. 0.89 | 1. 0.55-1.44 | 1. 0.6362 |
| 1. Fluid and electrolyte disorders | 1. 0.97 | 1. 0.66-1.41 | 1. 0.8704 |
| 1. Hypertension | 1. 1.28 | 1. 1.12-1.47 | 1. 0.0003 |
| 1. Hypothyroidism | 1. 0.91 | 1. 0.74-1.12 | 1. 0.3602 |
| 1. Liver disease | 1. 1.26 | 1. 0.94-1.69 | 1. 0.119 |
| 1. Other neurological disorders | 1. 1.27 | 1. 0.88-1.83 | 1. 0.2034 |
| 1. Obesity | 1. 1.32 | 1. 1.09-1.60 | 1. 0.0052 |
| 1. Chronic pulmonary disease | 1. 1.23 | 1. 1.04-1.46 | 1. 0.0175 |
| 1. Rheumatoid arthritis/collagen vascular diseases | 1. 1.29 | 1. 0.94-1.76 | 1. 0.1122 |
| 1. Substance-related disorders | 1. 1.35 | 1. 0.93-1.96 | 1. 0.1111 |
| 1. Solid tumor without metastasis | 1. 1.05 | 1. 0.75-1.49 | 1. 0.7654 |
| 1. Valvular disease | 1. 1.36 | 1. 0.99-1.88 | 1. 0.0566 |
| 1. **Pregnancy in year before depression diagnosis (no = 0, yes = 1)** | 1. 0.96 | 1. 0.72-1.27 | 1. 0.769 |
| 1. **Pregnancy in year after depression diagnosis (no = 0, yes = 1)** | 1. 1.80 | 1. 1.39-2.34 | 1. <0.0001 |
| 1. **Year of depression diagnosis. Baseline is 2008. (no = 0, yes = 1)** |  |  |  |
| 1. 2009 | 1. 1.42 | 1. 1.12-1.81 | 1. 0.0038 |
| 1. 2010 | 1. 1.74 | 1. 1.38-2.20 | 1. <0.0001 |
| 1. 2011 | 1. 1.85 | 1. 1.47-2.34 | 1. <0.0001 |
| 1. 2012 | 1. 1.66 | 1. 1.30-2.12 | 1. <0.0001 |
| 1. 2013 | 1. 1.68 | 1. 1.31-2.17 | 1. 0.0001 |
| 1. 2014 | 1. 2.04 | 1. 1.59-2.61 | 1. <0.0001 |
| 1. 2015 | 1. 2.12 | 1. 1.65-2.73 | 1. <0.0001 |
| 1. 2016 | 1. 2.63 | 1. 2.04-3.39 | 1. <0.0001 |

1. **Table S7. Results from sensitivity analysis on influence of imputing missing diagnoses for the work leave.**

| 1. **Reference Group** | 1. **Treatment Group (no = 0, yes = 1)** | 1. **Hazard Ratio** | 1. **95% Confidence Interval** | 1. **p-value** | |
| --- | --- | --- | --- | --- | --- |
| 1. Non-adherent to both antidepressant and psychotherapy treatment§ | 1. Adherent to either antidepressant or psychotherapy treatment§ | 1. 0.85 | 1. 0.77-0.92 | 1. 0.0002 | |
| 1. No antidepressant treatment | 1. Non-adherent to antidepressant treatment | 1. 1.19 | 1. 1.08-1.32 | 1. 0.0008 | |
|  | 1. Adherent to antidepressant treatment | 1. 1.11 | 1. 0.99-1.25 | 1. 0.0726 | |
| 1. Non-adherent to antidepressant treatment† | 1. Adherent to antidepressant treatment† | 1. 0.92 | 1. 0.82-1.04 | 1. 0.1777 | |
| 1. No psychotherapy treatment | 1. Non-adherent to psychotherapy treatment | 1. 0.94 | 1. 0.84-1.06 | 1. 0.3021 | |
|  | 1. Adherent to psychotherapy treatment | 1. 0.76 | 1. 0.66-0.86 | 1. <0.0001 | |
| 1. Non-adherent to psychotherapy treatment‡ | 1. Adherent to psychotherapy treatment‡ | 1. 0.81 | 1. 0.73-0.90 | 1. 0.0001 | |
| 1. § Analysis excludes patients without antidepressant or psychotherapy treatment 2. † Analysis excludes patients without antidepressant treatment 3. ‡ Analysis excludes patients without psychotherapy treatment | | | | |  |


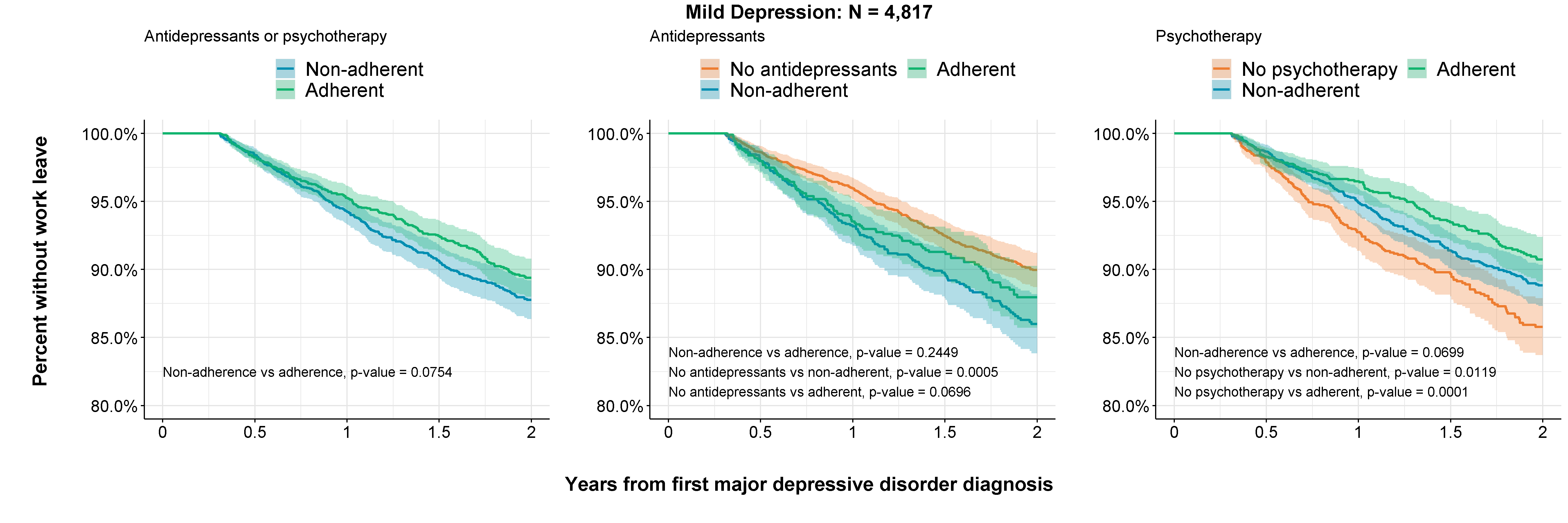

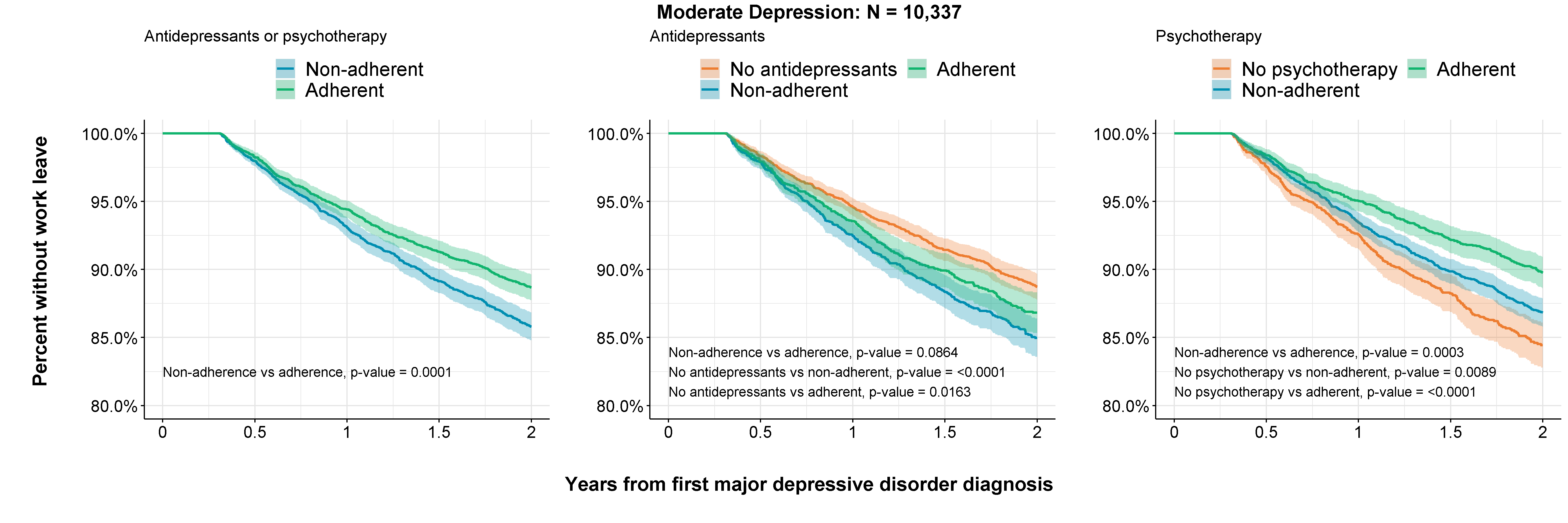

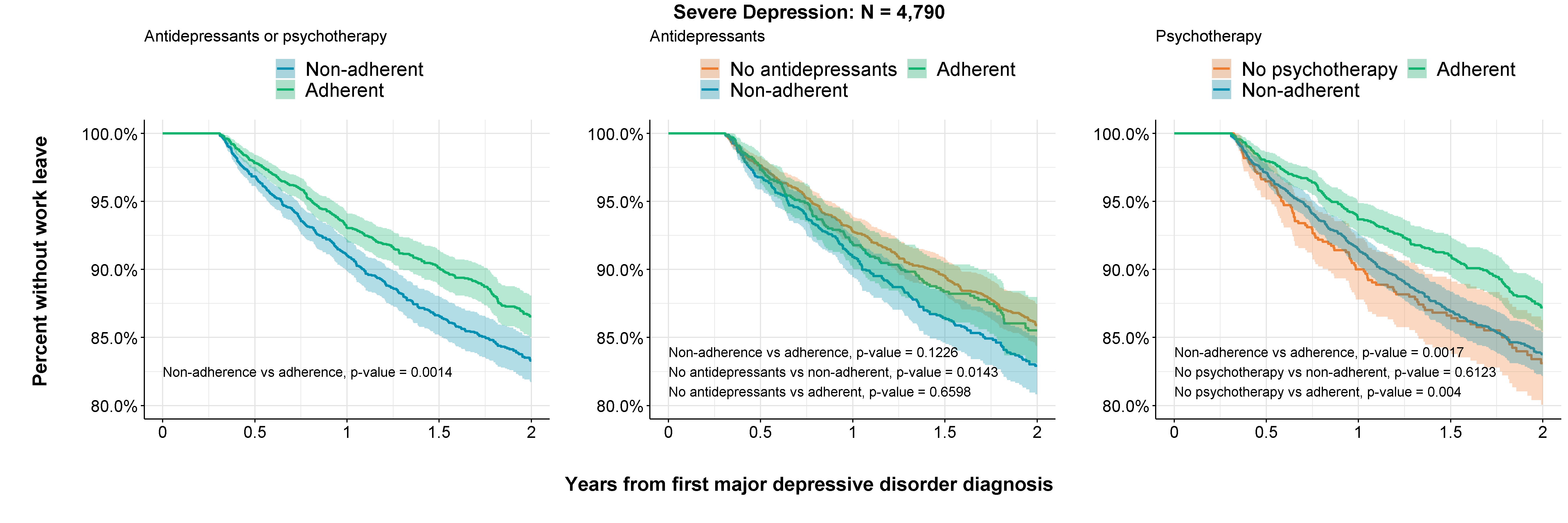


1. **Figure S1. Kaplan-Meier curves assessing the association between antidepressant and psychotherapy adherence with time to a non-pregnancy work leave- subset by MDD severity. P-values are calculated from chi-squared log rank tests between treatment groups.**
